# Heterozygous Single-Nucleotide Polymorphism Genotypes at Heat Shock Protein 70 Gene Potentially Influence Thermo-Tolerance Among Four Zebu Breeds of Nigeria

**DOI:** 10.3389/fgene.2021.642213

**Published:** 2021-04-12

**Authors:** Gbolabo Olaitan Onasanya, George Mutani Msalya, Aranganoor Kannan Thiruvenkadan, Chirukandoth Sreekumar, Gopalan Krishnaswamy Tirumurugaan, Adeboye O. Fafiolu, Matthew A. Adeleke, Abdulmojeed Yakubu, Christian Obiora Ndubuisi Ikeobi, Moses Okpeku

**Affiliations:** ^1^Department of Animal Science, Federal University Dutse, Dutse, Nigeria; ^2^Department of Animal Breeding and Genetics, Federal University of Agriculture Abeokuta, Abeokuta, Nigeria; ^3^Mecheri Sheep Research Station Pottaneri, Tamil Nadu Veterinary and Animal Sciences University, Chennai, India; ^4^Biotechnology Laboratory, Post Graduate Research Institute in Animal Sciences, Tamil Nadu Veterinary and Animal Sciences University, Chennai, India; ^5^Department of Animal, Aquaculture and Range Sciences, Sokoine University of Agriculture, Morogoro, Tanzania; ^6^Department of Animal Biotechnology, Madras Veterinary College, Tamil Nadu Veterinary and Animal Sciences University, Chennai, India; ^7^Department of Animal Nutrition, Federal University of Agriculture, Abeokuta, Abeokuta, Nigeria; ^8^Discipline of Genetics, School of Life Sciences, College of Agriculture, Engineering and Sciences, University of KwaZulu-Natal, Durban, South Africa; ^9^Department of Animal Science, Faculty of Agriculture, Nasarawa State University, Keffi, Nigeria

**Keywords:** heat tolerance, heterozygosity, native cattle, polymorphisms, thermal stress

## Abstract

Genetic variants at heat shock protein 70 gene and their influence on heat stress (HS) tolerance were studied among selected Nigeria zebu, namely, 25 White Fulani (WF), 21 Sokoto Gudali (SG), 21 Red Bororo (RB), and 23 Ambala (AM). Detection of single nucleotide polymorphism (SNP) followed by determination of genotype and genotypic frequency was made among the selected breeds. The heat tolerance coefficient (HTC) was determined from thermo-related parameters including body temperature, rectal temperature, and respiratory rate. Thermo-Tolerance was evaluated through the SNP–thermo-parameter relationship. Statistical analyses were done using the GLM procedure in SAS. A quantitative real-time/high-resolution melting-based assay detected twelve genetic variants. Five of these were common and shared across all breeds of cattle. Of the remaining seven variants, three were specifically identified in AM, two in SG, and two in RB. Also, SNPs were evaluated and four unique SNPs (C151T, C146T, G90A, and C219A) were identified. Heterozygous animals had lower HTC suggesting their potential to withstand HS than homozygous counterparts. The WF and RB animals had significantly lower values for all parameters (BT, RT, RR, and HTC) compared to AM and SG breeds. Thermo-related parameters were significantly different (*P* < 0.001), and it is recommended that screening of SNPs in zebu is needed to enable selection for improved thermo-tolerance.

## Introduction

Among livestock, cattle are the main contributors to animal proteins and a substantial proportion to the economies of many developing countries (Herrero et al., 2013). In recent years, the disruptive effects of global warming on livestock production systems and the attending negative impact on animal-protein source are quite profound, and Nigeria as a country is not left out. The impact of heat stress (HS) on cattle due to global warming can be overwhelming. According to West (2003), cows under HS exhibit reduced feed intake and decreased activity. The author further showed that under such situation animals increase the respiratory rate and peripheral blood flow leading to negative effects on both physiological status and production such as lowered milk quality and quantity.

Habeeb et al. (2018) have shown that an increase in ambient temperatures combined with relative humidity affects the ability of cattle to maintain homeothermy and, when the core body temperature is more than the normal physiological level, results in high HS. These negative impacts result in significant loss of income and increased management costs (Kishore et al., 2016). The impact of HS needs to be addressed and ameliorated to maintain animal health status, adaptability, survivability, and performance. It is therefore of great necessity that livestock species are bred to reproduce and produce normally under these circumstances (West, 2003). Before this is done, biological information including the genetic potential of various livestock species with respect to various traits of economic importance needs to be elucidated (Onasanya et al., 2020).

Heat shock proteins (*HSPs*) are a group of proteins conserved in prokaryotic and eukaryotic organisms and play vital roles in cell response to environmental stress (Morimoto and Santoro, 1998). The genes related to *HSP* including *HSP* 70 have been shown to play important roles in follicular development, embryonic survival, and pregnancy maintenance (Sagirkaya et al., 2006). These genes are also regarded as determinants of animals’ capability to tolerate and survive well under stressful physiological conditions and environments (Hansen, 2004). Thus, the functional characterization of members of the *HSP* gene family has been known to play an important role in the selection and development of breeding programs intended at obtaining animals that may continue to better perform under changing climatic conditions (Johnson, 2018). *HSP* 70 particularly is a subfamily of *HSP* genes, which has been found to play essential roles in cellular protection, immune response, protein synthesis, cyto-skeletal protection, protein folding and unfolding, protein translocation and regulation of steroid hormone receptors, transportation, refolding of protein, protection of proteins from cellular stress, inhibitory apoptosis, and adaptation during and after thermal assault in various livestock species including cattle (Wurst et al., 1989). It is a powerful candidate marker for health, reproduction, and productive traits (Wurst et al., 1989). The gene is located at 23q13 in the bovine genome, a close proximity to the major histocompatibility complex (MHC), which plays important roles in immune responses and combating infectious diseases in cattle (Fries et al., 1986; Anderson and Davies, 1994).

Detection of genetic variants in the *HSP* 70 gene in various livestock species as well as in humans have increased in recent years (Xiong et al., 2013). Polymorphisms in this gene and the resultant phenotypic traits can potentially be exploited for determination of tolerance or resistance of livestock species to thermal stress. For instance, polymorphisms can be used to drift herds toward superior thermo-tolerance ability through the improvement of heat-vulnerable stocks with thermo-tolerant stocks. Also, genetic variants could be important for developing and managing livestock in the face of climate change (Monua et al., 2020), more efficient resource utilization, and improved production performance in terms of fertility, milk production, feed intake, growth, conception rates, and animal health (Bhat et al., 2016).

High-resolution melting (HRM) is a post-polymerase chain reaction (PCR) analysis technique, capable of discriminating DNA sequences based on composition, length, GC content, or strand complementarity (Reed et al., 2007). It is a simple and fast technique based on PCR melting (dissociation) curve techniques, enabled through improved double-stranded DNA (dsDNA)–binding dyes along with next-generation real-time (RT) PCR instrumentation and analysis software (Reed et al., 2007). The combined usage of high-resolution melting analysis (HRMA) with quantitative real-time (qRT)-PCR permits an efficient and rapid genotyping and detection of polymorphism in dsDNA (Gori et al., 2012). This technique generates DNA melting curve profiles that are very specific and sensitive enough to distinguish sequence variation, enabling mutation scan, methylation, and genotyping analysis (Gori et al., 2012). In the last few years, an increasing number of research articles have been published on qRT-PCR/HRMA-based assays, especially in the identification of human (Wynyard et al., 2011) and animal pathogens (Erdem et al., 2016). The evolution of qRT-PCR/HRMA-based assays involving the use of fluorescent intercalating dye (SYBR Green) with a new generation of light cyclers is a high-throughput technology with an established average temperature range of 66–96°C for mutation analyses, genetic variant analyses, polymorphism study, and single-nucleotide polymorphism (SNP) genotyping in large populations, which has emerged as cutting-edge technology with high efficiency and cost-effectiveness. This technology is gradually becoming a technique of choice for rapid genotyping and study of polymorphisms (Gori et al., 2012).

Previous studies in zebu cattle breeds suggested that they are superior in adapting to tropical climatic conditions compared to their European counterparts (Hansen, 2004). Zebu animals are naturally adapted to a wide range of agro-climatic conditions (Bhat et al., 2016) and possess varying levels of tolerance to humidity-related diseases and poor nutrition (Bhat et al., 2016). However, the scientific basis of the strength of these animals has not been studied explicitly.

Nigeria is among the tropical countries with severe influence of thermal stress that negatively affects production performance of livestock. Nigerian native (indigenous) cattle breeds are predominantly zebu, some of which are particularly adapted to the Northern part of the country with the highest heat assault. However, very little effort has been made to understand their HS tolerance status. On the other hand, genetic variants including SNPs in the bovine *HSP* genes including *HSP* 70, 90, and 90AB1 have been shown to influence thermo-tolerance capability in various cattle populations (Deb et al., 2013; Bhat et al., 2016; Onasanya et al., 2020).

The heat shock protein subfamily consists of facultative genes whose expressions are largely influenced by changes in environmental temperatures. Onasanya et al. (2020) reported the potential of H*SP* 90 as strong candidate genes for possible biomarkers for measuring thermal assault. However, the *HSP* 70 gene is both a constitutive and facultative gene and is more sensitive to thermal signals (Bhat et al., 2016; Kishore et al., 2016). The present study was set out to test the hypothesis that *HSP* 70 possesses equal sensitivity as *HSP* 90 as candidate gene for thermal assault determination.

Various physiological parameters including body temperature (BT), rectal temperature (RT), respiratory rate (RR), and panting score (PS) have been evaluated in livestock and associated with the capability of animals to withstand HS (Richard et al., 2020). In the present study, BT, RT, and RR were evaluated from which the heat tolerance coefficient (HTC) was calculated. Various factors including changes in the environment such as increased air temperature affect the BT of cattle and lead to production loss after several thermal assaults (Habeeb et al., 2018). It is of great importance that BT of cattle is monitored and maintained during HS. The RT is also an important factor considered when the adaptability index in hot regions is considered. A high value of RT is an indication that the animal heat dissipation mechanisms fail to maintain homeothermia (McManus et al., 2009). Furthermore, animals exhibiting increased RR demonstrate increased peripheral blood flow and sweating, leading to deleterious effects on both production and physiological status of the animals (West, 2003). It is therefore of great necessity that RR is measured and included in analyses to understand the tolerance of animals to HS and; for this reason, the parameter was included in this study.

On the other hand, the HTC is a good indicator of adaptability of the animals (Benezra, 1954). The association between the *HSP* 70 polymorphisms and heat tolerability parameters have been detected in various zebu cattle breeds (Bhat et al., 2016). Various genotypes including heterozygous SNP genotypes were shown to confer better thermo-tolerance in cattle (Lamb et al., 2007). The objectives of the study were, therefore, firstly to examine the polymorphisms of the *HSP* 70 gene using qRT-PCR/HRMA-based assay to characterize the genetic variations and, secondly, to detect SNPs and the related SNP genotypes at the *HSP* 70 gene to body parameters, with the intent to establish thermo-tolerance potentials in Nigerian zebu cattle breeds.

## Materials and Methods

### Study Animals and Sampling

The study animals were adult bulls (total 90) from four extant breeds of Nigerian zebu: 25 White Fulani (WF), 21 Sokoto Gudali (SG), 21 Red Bororo (RB), and 23 Ambala (AM) were randomly sampled while awaiting slaughter (in government-approved slaughterhouses and abattoirs) in the study area. The animals were brought to the slaughterhouses from 10 states of northern Nigeria, namely, Sokoto, Kebbi, Zamfara, Katsina, Kano, Kaduna, Jigawa, Bauchi, Gombe, and Yobe. The animals are traditionally managed in the communal rangelands by their owners. Care was taken to maintain heterogeneity as much as possible. Animals with similar clear breed characteristics and relatedness as earlier determined by various researchers, e.g., Katie and Alistair (1986) and Williamson and Payne (1990), were sorted into groups and randomly sampled. Important information needed from each animal such as age and breed was obtained from the farmers and herd owners before sampling. Based on the information shared by farmers, characteristics of animals, availability, and scientific recommendation by previous researchers, we regarded the animals at the slaughterhouse for each of the four zebu breeds as homogenous and therefore only 90 animals were sampled. For example, Li et al. (2020) and Nazareno et al. (2017) hypothesized and affirmed that a minimal sample size between 3 and 20 is sufficient to estimate genetic diversity studies.

### DNA Purification

The DNA extraction was done using the HiPurA^TM^ Multi-Sample DNA Purification (MolBio^TM^ Himedia^®^, Mumbai, India) kit, following the manufacturer’s protocols. Quality and quantity check was done using a Thermo Scientific-NanoDrop 2000 spectrophotometer (Shimadzu Corporation, Kyoto, Japan) and stored at −20°C. The DNA purification was conducted at the Biotechnology Laboratory, Post Graduate Research Institute in Animal Sciences, Tamil Nadu Veterinary and Animal Sciences University, Chennai, India. All incubations were done on an ACCUBLOCK^TM^ digital dry bath (Labnet International, Edison, NJ, United States), whereas all centrifuge processes were done in a HiElute Miniprep spin column using a Thermo Scientific Nanofuge (MCROCL 21/21R) micro-centrifuge (Waltham, MA, United States).

### Polymerase Chain Reaction and Amplification Condition

The polymerase chain reaction was carried out in a total volume of 15 μl comprising of template DNA 1.0 μl, 1.0 μl of each of forward and reverse primers (F-AAACATGGCTATCGGCATCGACCT and R-AGGCTTGTCTCCGTCGTTGATGA; Bhat et al., 2016), 7.5 μl PCR Master Mix (2×) (GeNei^TM^ Red Dye PCR Master Mix), and 4.5 μl of nuclease-free water. Amplification was performed in a TaKaRa Thermal Cycler Dice^TM^ version III (Takara Bio Inc., Shiga, Japan). The amplification condition involved initial denaturation at 94°C for 5 min, followed by 45 cycles of denaturation at 94°C for 60 s, annealing temperature of 65°C for 45 s, and extension at 72°C for 1 min. Final extension was made with 7 min at 72°C. The PCR products were isolated on 2% agarose gel electrophoresis after staining with 1 μg/ml of ethidium bromide and visualized under Bio-RAD Gel Doc^TM^ XR+ Imaging System version 5.1 (Gel Documentation Molecular Imager, Bio-Rad Laboratories, Inc., CA, United States). The gel bands were then excised carefully and placed into sterilized vials and stored at −20°C. Thereafter, the excised gel bands were digested manually after a series of centrifugations for 5 min at 10,500 rpm. A layer of supernatant (purified DNA) was formed on the surface of the gel, and the purified supernatant (DNA fragment) was treated as DNA template for subsequent qRT-PCR/HRMA-based assays.

### Quantitative Real-Time PCR and High-Resolution Melting Analysis-Based Assays

The quantitative real-time PCR and HRMAs were carried out to identify and detect possible genetic variants of *HSP* 70 in the samples. The qRT-PCR reactions were carried out on a total volume of 20 μl containing a purified DNA fragment (template DNA) 1.0 μl, 1.0 μl of each of forward and reverse primers, SYBR Green Master Mix (2×) of 10.0, and 7.0 μl of nuclease-free water. The qRT-PCR amplification was performed in a Roche LightCycler^®^ 96 with software version 1.01.01.0050 (Roche Diagnostics, Mannheim, Germany). The qRT-PCR amplification condition (with HRMA option) comprised of a preincubation step of 5 min, followed by 45 cycles of denaturation at 95°C for 10 s, annealing at 65°C for 10 s, and extension at 72°C for 10 s. Specifically, the HRMA was performed by first heating to 95°C for 1 min, cooling to 37°C for 30 s, heating to 65°C for 1 s, and then melting with continuous acquisition (15 readings/°C) of the florescence signal up to 97°C. Genetic variants of the *HSP* 70 gene generated across breeds of Nigerian *Bos* (*B*.) *indicus* were analyzed using Roche HRM Software version 1.1.0.1320. Individual samples (PCR products) were differentiated into distinct genetic variants via high-resolution melting curve profiles (derivative HRM curve/dissociation curve, differential plot, and normalized melt curves) as depicted with distinct SYBR green (dye) fluorescence after the methods of Gori et al. (2012) and Chambliss et al. (2017).

### Sequencing and Detection of Single-Nucleotide Polymorphisms

The polymerase chain reaction products were purified and subsequently sequenced using an automated ABI DNA Sequencer (Eurofins Genomics Pvt. Ltd., Bangalore, India). The nucleotide sequences were visualized and edited by chromatogram analyses on a SeqMan Ngen Tool (DNASTAR^®^, Inc., Madison, WI, United States). We aligned our sequences with that of unrelated bovine *HSP* 70 nucleotide sequence (NCBI Ascension No: U09861) obtained from the NCBI GenBank database to detect SNP variabilities. A sequence map showing a unimodal peak indicated the homozygous genotype, and the overlapping peaks indicated heterozygous genotype and the total genotypes were obtained by direct counting. The SNP genotypes generated were associated with thermo-related measurements to unravel the potential ability of the animals to withstand the negative effects of high heat.

### Assessment of Tolerance to Heat Stress and Statistical Analyses

The body temperature and RT were measured using a digital thermometer while RR was measured using a stethoscope. The measurements were taken on each for each of the extended 30 sampling days with the aim of obtaining variation in the animals. Sampling duration was 6 h between 9 am and 3 pm when the average ambient temperature was 37.5°C and humidity was 24%. Wind speed in the lairages and surrounding areas ranged between 3.5 and 4.5 miles per hour whereas the wind chill temperature was 35.5°C. Then, these parameters were analyzed to obtain averages which were used to calculate HTC values (measure of adaptability of animal assaults of thermal stress) using a Linear Equation (1) according to Benezra’s coefficient of adaptability (BCA) in Benezra (1954).

(1) HTC=RR⁢/⁢23±RT⁢/⁢38.33

where RR, respiratory rate (bpm); RT, rectal temperature (°C), normal rectal temperature (38.33°C), and normal respiratory rate (23 bpm). The essence of rectal temperature as used in this formula tends to be a more accurate measurement of body temperature than oral and axillary temperature readings; therefore, it is taken as a measure of internal temperature that reflects and gives a more accurate body temperature (Susan et al., 2019).

Thereafter, statistical analyses were performed using the generalized linear model (GLM) procedure of the statistical analysis system (SAS) PROPRIETARY Software Version 9.2 (SAS Institute Inc., NC, United States) followed by Duncan’s multiple-range test to separate the means. The model (2) involved parameters BT, RT RR, and HTC as dependent variables whereas SNP genotypes and breeds were the fixed variables. *P*-values <0.05 were considered significant. Finally, the Hardy–Weinberg equilibrium (HWE) for genotypic frequency was analyzed with goodness of fit and tested using the Chi-square (χ^2^) test.

(2) $$Y_{j}=m+S_{i}+B_{j}+E_{i\,j}$$

where

*Y*_*ijk*_ = observation of the thermo-related measurements;

μ = overall mean;

*S*_*i*_ = effect of the *i*th genotypes on thermo-tolerance measurements;

*B*_*j*_ = effect of the *j*th breed on thermo-tolerance measurements traits; and

*E*_*ij*_ = random error associated with *Y*_*ij*_ observations for thermal-related measurements of the zebu cattle breeds.

## Results

### The High Resolution Melting Analysis Assays and Genetic Variants at *HSP* 70 Gene in the DNA Samples

In this study, detection of variations at *HSP* 70 among our DNA samples has been made by looking at the different melting temperatures shown by 12 distinct SYBR Green fluorescence (dye), viz., Purple (PRP), Red (RED), Orange (ORG), Green (GRN), Lemon (LMN), Brown (BRN), Chocolate (CHO), Yellow (YLO), Magenta (MGT), Blue (BLU), Army green (AGN), and Navy blue (NBL), and the respective melting temperatures are presented in Table 1.

**TABLE 1:** Heat shock protein (*HSP*) 70 gene variants/alleles at different melting peaks.

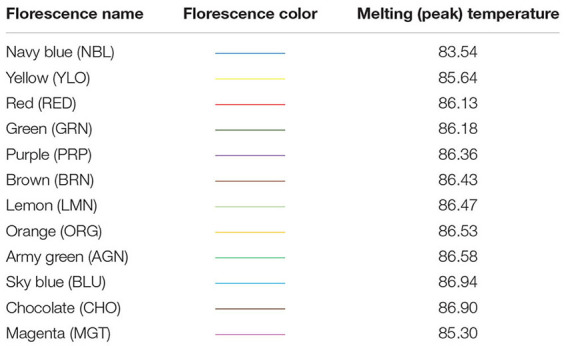

**FIGURE 1:**
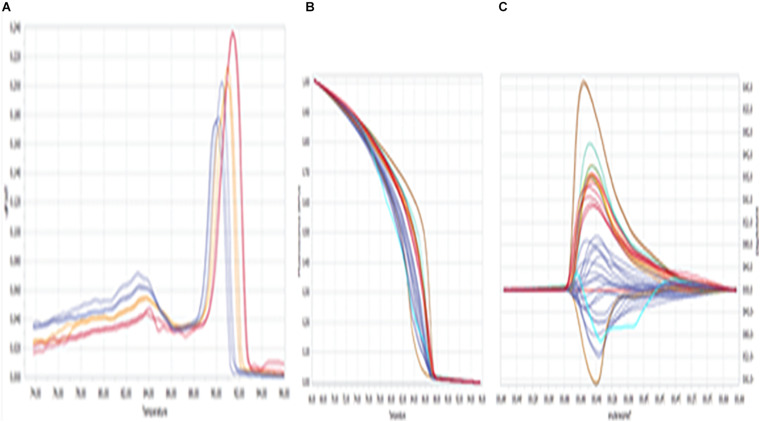
High-resolution melting (HRM) curve profiles. **(A)** Dissociative/derivative HRM curves; **(B)** normalized melt curves; **(C)** differential plot curves at *HSP* 70. Delta Tm discrimination is 50%, and curve shape discrimination is 50%.

The fluorescence (difference in melting temperature) was considered to be a genetic variant among the samples and was confirmed by various HRM curve profiles, and the same was summarized in Figure 1. The profiles included a dissociative/derivative HRM curve with distinct SYBR Green dye fluorescence due to differences in melting (peak) temperatures among the DNA samples (Figure 1A), a normalized melt curve showing dissociation of the double strand of *HSP* 70 and rhythm of co-mingled/intercalating dye (SYBR Green) fluorescence (Figure 1B), and the differential plot curve (Figure 1C).

The distribution of variants in the samples (or in the animal breeds) including percentages of each is presented in Table 2. The highest variant was the PRP which occurred in 41.8% of the samples followed by RED (16.4%) while the lowest-occurring variants were BRN, CHO, YLO, MGT, BLU, AGN, and NBL which comprised 1.8% of the genetic variants each (Table 2). Two variants (PRP and ORG) were detected in DNAs of all animal breeds involved in this study. The LMN occurred in three out of four breeds while RED and GRN each occurred in two populations. Combined, the total number of variants was 110.

**TABLE 2:** Detection of genetic variants at *HSP* 70 gene in Nigerian native cattle breeds.

**SN**	**Genetic variants**	**Frequency**	**Breeds**	**Distribution (%)**
1	PRP	46	WF, AM, SG, RB	41.8
2	RED	18	WF, SG	16.4
3	ORG	18	WF, AM, SG, RB	16.4
4	GRN	6	AM, RB	5.5
5	LMN	8	WF, AM, RB	7.3
6	BRN	2	AM	1.8
7	CHO	2	SG	1.8
8	YLO	2	RB	1.8
9	MGT	2	RB	1.8
10	BLU	2	AM	1.8
11	AGN	2	AM	1.8
12	NBL	2	SG	1.8
Total	110		100	

*PRP, purple; RED, red; ORG, orange; GRN, green; LMN, lemon; BRN, brown; CHO, chocolate; YLO, yellow; MGT, magenta; BLU, blue; AGN, army green; NBL, navy blue; WF, White Fulani; AM, Ambala; SG, Sokoto Gudali; RB, Red Bororo.*

We arranged the variants to obtain the distribution among the four breeds and observed that there were more variants (7) in AM samples followed by RB (6) and SG (5) and least (4) in WF (Table 3).

**TABLE 3:** Distribution of genetic variants of *HSP* 70 gene within native cattle breeds of Nigeria.

**Breed**	**Genetic variants**	**Occurrence of variants per breed**
WF	PRP, RED, ORG, LMN	4
AM	PRP, ORG, GRN, LMN, BRN, BLU, AGN	7
SG	PRP, RED, ORG, CHO, NBL	5
RB	PRP, ORG, GRN, LMN, YLO, MGT	6

*PRP, purple; RED, red; ORG, orange; GRN, green; LMN, lemon; BRN, brown; CHO, chocolate; YLO, yellow; MGT, magenta; BLU, blue; AGN, army green; NBL, navy blue; WF, White Fulani; AM, Ambala; SG, Sokoto Gudali; RB, Red Bororo.*

### Identification of SNPs Within *HSP* 70 Gene in Four Breeds of Nigerian Zebu Cattle

**FIGURE 2:**
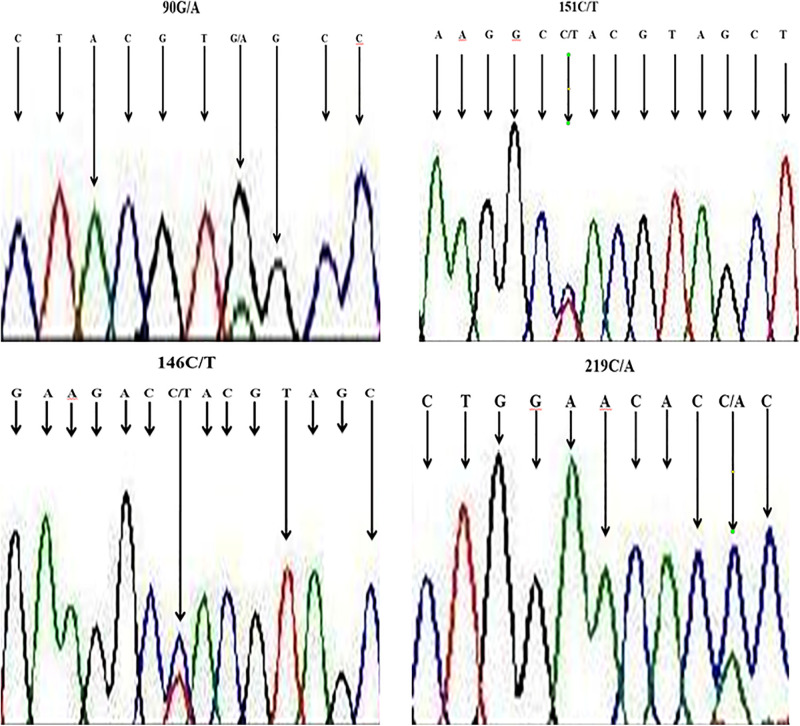
Section of sequence map at *HSP* 70 showing sites with base exchange (SNP) in DNA samples of Nigerian zebu cattle.

We aligned nucleotide sequences of our samples (*B*. *indicus*) with sequences of a similar region in *B*. *taurus* (obtained from NCBI), and SNPs were revealed at four locations, viz., locations 90 (from G to A), at site l46 and 151 change (from C to T), as well as at site of sequences 219 with change from C to A. The sequencing map showing a unimodal peak indicated the homozygous genotype, and the overlapping peaks indicated heterozygous genotype (Figure 2).

Notably, the four SNPs were specific in the cattle breeds, i.e., one SNP was detected in the DNA of each of the breeds (Table 4). The resulting SNP genotypes are also listed in Table 4.

**TABLE 4:** Single nucleotide polymorphisms (SNP) genotype within exon 1 of *HSP* 70 gene in four Nigerian Zebu cattle breeds.

**Breed**	***N***	**SNP location**	**SNP code**	**Genotypes**		
WF	25	151	C > T	CC (5)	CT (14)	TT (6)
AM	23	146	C > T	CC (7)	CT (5)	TT (11)
SG	21	90	G > A	GG (9)	GA (7)	AA (5)
RB	21	219	C > A	CC (4)	CA (10)	AA (7)

Among the four SNPs, three (C151T, C146T, and G90A) were transitions causing base change from purine to purine, whereas one (C219A) was transversion, i.e., causing change of a pyrimidine base to purine base (Tables 5, 6). Furthermore, one SNP was classified as synonymous, one whose amino acid does not cause any subsequent changes in translated protein product, whereas three others were non-synonymous, ones which cause changes to subsequent translated protein products (Tables 5, 6).

**TABLE 5:** Characteristics of SNP variants identified in the present study.

**Breed**	**SNP variants**	**Nature of SNP variants**	**Changes in protein products**	**Types of mutation**
WF	C151T	Transition	Serine > leucine	Non-synonymous
AM	C146T	Transition	Proline–proline	Synonymous
SG	G90A	Transition	Alanine > arginine	Non-synonymous
RB	C219A	Transverse	Cysteine > Threonine	Non-synonymous

**TABLE 6:** Genotypic and allelic frequencies of SNPs identified in the present study.

**Breed**	**SNP loci**	**SNP genotypes**	**Genotypic frequency**	**Allele**	**Allelic frequency**	**HWE test χ^2^**	***P*-value (*P*)**
White Fulani	151C > T	CC	0.20	CT	0.48	0.102	0.749 (*P* > 0.05)
		CT	0.60		0.52		
		TT	0.24				
Ambala	146C > T	CC	0.30	CT	0.44	7.125	0.008 (*P* < 0.05)
		CT	0.22		0.56		
		TT	0.48				
Sokoto Gudali	90G > A	GG	0.43	GA	0.60	2.333	0.127 (*P* > 0.05)
		GA	0.33		0.40		
		AA	0.24				
Red Bororo	219C > A	CC	0.19	CA	0.43	0.057	0.810 (*P* > 0.05)
		CA	0.48		0.57		
		AA	0.33				

Furthermore, the genotypic and allelic frequencies of SNPs were computed. Frequencies were variable in each group and no clear pattern of the genotypes; the three SNPs (C151T, G90A, and C219A) were in agreement with the HWE (*P* > 0.05), and one (C146T) was not (*P* < 0.05).

### Thermo-Tolerance Parameters and Their Association With SNP Genotypes

With respect to body parameters, i.e., BT, RT, RR, and HTC, our data were rearranged such that the parameters were related to the SNP genotypes as well as to the animal breeds. The range values for these parameters were from 38.180 ± 0.01 to 39.952 ± 0.10°C for BT, 38.235 ± 0.30 to 39.900 ± 0.01°C for RT, 102.246 ± 0.21 to 108.110 ± 0.20 bpm for RR, and 5.154 ± 0.01 to 6.950 ± 0.01 for HTC (Table 7). In general, the BT values were low in the heterozygote CT animals (38.186 ± 0.10°C) compared to homozygotes CC and TT animals at the 151 site. Likewise at the 90 SNP site, the heterozygote animals (GA genotype) had the lowest BT (38.190 ± 0.01°C) compared to both homozygotes (AA and GG genotypes). A similar situation was observed at the 219 site, where the heterozygote animals (CA genotype) had the lowest BT (38.180 ± 0.10°C) than both homozygote animals (AA and CC genotypes). The RT values showed a similar trend as those of the BT values, and therefore, animals possessing the heterozygous genotypes had lower RT values than the homozygous animals at the three sites (Table 7). It was noted that the RR and HTC values showed a similar trend as those of BT and RR values.

**TABLE 7:** Mean (±SE) values of heat related parameters in the study animals in relation to three SNP sites of *HSP* 70.

**SNP site**	***SNP genotypes/*breeds	**Body temperature (°C)**	**Rectal temperature (°C)**	**Respiratory rate (bpm)**	**Heat tolerance coefficient**
151	CC	39.838 ± 0.20^a^	39.713 ± 0.16^a^	107.071 ± 0.35^a^	5.715 ± 0.01^a^
	CT	38.186 ± 0.10^c^	38.235 ± 0.30^c^	102.246 ± 0.21^c^	5.154 ± 0.01^c^
	TT	38.886 ± 0.11^b^	38.717 ± 0.10^b^	103.450 ± 0.21^b^	5.690 ± 0.01^b^
90	GG	39.952 ± 0.10^a^	39.900 ± 0.01^a^	108.110 ± 0.20^a^	6.950 ± 0.01^a^
	GA	38.190 ± 0.01^c^	38.273 ± 0.10^c^	102.431 ± 0.10^c^	5.254 ± 0.10^c^
	AA	38.894 ± 0.01^b^	38.810 ± 0.18^b^	103.556 ± 0.01^b^	5.525 ± 0.01^b^
219	CC	39.712 ± 0.10^a^	39.720 ± 0.05^a^	107.100 ± 0.20^a^	5.655 ± 0.01^a^
	CA	38.180 ± 0.01^c^	38.240 ± 0.10^c^	102.250 ± 0.10^c^	5.324 ± 0.10^c^
	AA	38.884 ± 0.01^b^	38.712 ± 0.04^b^	103.524 ± 0.01^b^	5.415 ± 0.01^b^
	WF	38.243 ± 0.23^c^	38.343 ± 0.842^b^	102.929 ± 0.56^c^	5.475 ± 0.03^c^
	AM	38.633 ± 0.24^b^	38.544 ± 0.84^a^	103.533 ± 0.60b	5.507 ± 0.03^b^
	SG	38.925 ± 0.30^a^	35.841 ± 0.08^c^	104.350 ± 0.74^a^	5.550 ± 0.04^a^
	RB	38.269 ± 0.17^c^	38.221 ± 0.60^b^	102.805 ± 0.41^c^	5.470 ± 0.02^c^

^*a,b,c*^Means with different superscripts within the same column are significantly different (*P < 0.05) for breeds of cattle, (***P < 0.001) for SNP genotypes.

These parameters (BT, RT, RR, and HTC) were evaluated with respect to the breeds of cattle used in the present study, and it was found that the WF and RB animals had significantly lower values for all parameters (BT, RT, RR, and HTC) compared to AM and SG breeds. On the other hand, the SG animals showed the highest values for all parameters and the values were statistically significant (*P* < 0.05) among the breeds.

The association between SNP genotypes and heat-related parameters revealed that the SNP genotypes at polymorphic locus C151T within the exon 1 region of the *HSP* 70 gene were associated with the thermo-tolerance traits in the WF breed and the results showed a significant effect (*P* < 0.001) on the considered thermo-tolerance traits.

## Discussion

The quantitative real time-PCR/HRMA-based assay has become an emerging and cutting-edge technology with high efficiency and cost-effectiveness in genotyping of SNPs in large populations. This technology uses fluorescent intercalating dye (SYBR Green) with a new generation of light cyclers which is a high-throughput technology with an established average temperature range of 66–96°C, which enables analyses of mutation and evaluation of polymorphisms in DNA samples, making it a technique of choice for such studies (Gori et al., 2012). Using these technologies, genetic variants have been detected in grapevine leaf roll-associated associated virus (Bester et al., 2012), in *Pseudomonas savastanoi* and Pathovars (Gori et al., 2012), and in multiple detection of genetic variants of *Mycobacterium leprae* drug resistance mutations and strain types (Li W. et al., 2011). In addition, the qRT-PCR/HRMA-based assay was used in the identification of human and animal pathogens (Wynyard et al., 2011), genotyping of drug-resistant bacteria isolates (Hoek et al., 2008), and human genetic variants linked to cancer (Krypuy et al., 2007).

The present study utilized the technology in the evaluation of polymorphisms of *HSP* 70 gene in Nigerian Zebu through which 12 genetic variants were detected. Polymorphisms at the *HSP* 70 gene in DNA samples of our Zebu suggested evidence of genetic diversity among them at this locus, and our results corroborate the findings of previous researchers (Hoek et al., 2008; Wynyard et al., 2011). The variability of the *HSP* 70 gene in Nigerian native cattle breeds suggests endowed ability to survive and adapt to a wide range of agro-climatic conditions and variations in assaults of environmental stress peculiar to tropical conditions (Bhat et al., 2016). This will be helpful in the population-based selection program of thermo-tolerant stocks for breed improvement programs and genetic conservation.

Similarly, in the present investigation four SNP loci within the coding region of *HSP* 70 in exon 1 were detected, three of which were transitions (C151T in WF, C146T in AM, G90A in SG), whereas one was transversion (C219A in RB). The detection of these SNPs is an indicative of polymorphism at the gene among the Nigerian native cattle breeds. The results of this work agreed with those of previous reports of Lamb et al. (2007) who detected eight SNPs in different cattle breeds and Bhat et al. (2016) who reported two SNPs (G > T and G > C) at site 149 in the Indian Tharparkar breed. Other researchers including Nishant et al. (2016) and Nikbin et al. (2014) have reported polymorphisms (two SNP loci) of the *HSP* B8 gene in the Indian Sahiwal breed and two SNP variants within the nucleotide sequence of *HSP* 70 in Boer goats and their crosses. Furthermore, SNPs within the coding region of *HSP* 70 have been reported in Chinese Holstein cattle (five novel SNPs) by Li Q. et al. (2011) and in Frieswal crossbred cattle (four SNPs) by Deb et al. (2013). Also, Sodhi et al. (2013) reported high variability of *HSP* 70 (54 SNPs) in three breeds of Indian Zebu and riverine Buffalo. In boars, Huang et al. (2002) detected five SNP sites within *HSP* 70. The implication of the variability at *HSP* 70 in Nigerian local cattle is the differences among them in relation to thermo-tolerance capability (Lamb et al., 2007), and this calls for selection and breeding for this trait of the animals.

The heat shock protein subfamily includes *HSP* 70 and *HSP* 90 genes which are facultative genes whose expressions are largely influenced by changes in environmental temperatures. However, the *HSP* 70 gene is both a constitutive and facultative gene which is very sensitive to little or no thermal signals from environmental thermal assault/surrounding temperature and more sensitive than other *HSP* genes (Sodhi et al., 2013; Kishore et al., 2016). Onasanya et al.’s (2020) study with *HSP* 90 reported that this gene could serve for molecular screening of animal population for potential thermo-tolerance and supported the claim of Bhat et al. (2016) and Kishore et al. (2016). However, Sodhi et al. (2013) claimed that *HSP* 70 is a more sensitive thermo-regulator candidate gene. Onasanya et al. (2020) reported the significance of the *HSP* 90 gene on thermo-tolerance potentials of Nigerian breeds of zebu cattle and found that the *HSP* 90 gene significantly lowered BT, RT, RR, and HTC; the results inspired this current study.

Regarding SNP genotypes of the *HSP* 70 gene and their association to thermo-tolerance capability among our animals, we noted that the presence of polymorphic loci 151C > T (WF breed), 90G > A (SG breed), and 219C > A (RB breed) within the coding region of exon 1 of *HSP* 70 had influence on the animals (lower thermo-tolerance). Different genotypes of the gene have been shown to reduce the capability of thermo-tolerance in Holstein cattle (Xiong et al., 2013). In the present study, SNP genotypes (CT in WF, GA in SG, and CA in RB) were possibly the ones resulting to lower values of BT, RT, RR, and HTC in heterozygous animals. This is suggestive of better thermo-tolerance and survivability under assaults of thermal condition than their homozygous counterparts (Nishant et al., 2016). The lower BT, RT, and RR indicated the ability of animals to cope under heat stress (Beatty et al., 2006).

Lower thermo-tolerance values reported in this study confirmed the earlier findings of Deb et al. (2013) who reported that favorable SNP genotypes of *HSP* 70 had evident influence on thermo-tolerance traits of Frieswal crossbreeds and Bhat et al. (2016) who identified the influence of SNPs at *HSP* 70 to confer better thermo-tolerance and survivability advantage on Indian Tharparkar cattle. Furthermore, lower values of HTC in our animals are in consonance with the reports of earlier works in Thai native cattle (Charoensook et al., 2012) and Sahiwal cattle (Sajjanar et al., 2015) and Frieswal cattle in India (Deb et al., 2013). In these studies, higher HTC indicated poor adaptability and inability to cope with stressful thermal condition.

In the present study, the WF and RB breeds had lower BT and RR than SG and AM breeds. The differences could be possibly due to inherent breed differences among these animals as suggested by Hansen (2004). Phenotypically, the WF breed are known for their predominant white coat color, which has been implicated in thermo-regulation while the majority of animals in the RB breed have been found to have thin skin type compared to AM and SG breeds which are predominantly thick skinned, a critical factor for thermo-regulation. Thermal conduction and convention is greatly affected by coat color and thickness and thinness of the skin. Thin-skinned animals have better thermal convection advantage over thick-skinned counterparts. Thus, lower BT and RR in this study for WF and RB breeds indicated better thermo-tolerance and adaptability relative to SG and AM breeds (Nishant et al., 2016) and possible vulnerability of the latter to thermal assault/stressful environmental conditions (Beatty et al., 2006). Elevated RR is a very important thermo-regulatory response to thermal stress which helps in heat dissipation via evaporative cooling metabolism (Beatty et al., 2006). Panting is an easiest method to evaluate the strength of the animal under intense thermal stress, and this occurs to increase cooling of the body by elevated respiratory evaporation.

Elsewhere, several reports have found SNPs at *HSP* 70 to have association with other traits in animals including for instance semen quality traits in boars (Huang et al., 2002), sperm characteristics in bulls including sperm-binding oviductal proteins, which increases longevity and viability of sperm in bulls and boars (Elliott et al., 2009), and calving traits in cattle (Rosenkrans et al., 2010). The lack or knockout of the *HSP* 70 gene led to a significant increase in apoptosis (Dix and Hong, 1998). Govin et al. (2006) found an evidently remarkable association between thermo-regulatory functions of *HSP* 70 and spermatid DNA-packaging proteins during spermatogenesis.

Finally, this study identified amino-acid changes in the translated protein due to single-nucleotide changes at the detected polymorphic loci of *HSP* 70. The positional base changes that resulted in changes in amino acid subsequently brought about changes in the protein product and functions of the favorable SNPs. These observations followed a similar trend with the findings of Lamb et al. (2007) who reported that positional base changes (G203C) in *HSP* 70 resulted in amino acid changes from glycine to alanine in Chinese Holstein which leads to changes in functional capacity of favorable SNPs. Furthermore, our work confirmed the earlier works of Rosenkrans et al. (2010) who reported that single base substitutional mutations in *HSP* 70 resulted in functional changes in the corresponding protein product in crossbred Brahman cows and they were implicated for better thermo-tolerance.

Heat stress is one of the critical factors among various environmental stressors that impede profitable livestock rearing. Our results elucidated 12 genetic variants of *HSP* 70 in Nigerian zebu detected through multiplex qRT-PCR-HRMA-based assay. Also, four SNP variants were detected at *HSP* 70 in our samples. The present investigation revealed that the animals with heterozygous SNP genotypes of *HSP* 70 remarkably had lower values of BT, RT, RR, and HTC, and this is indicative of better thermo-tolerance, survivability, and ability to cope under stressful thermal conditions. The elevated HTC index indicates poor adaptability/ability to cope under thermal environmental stress. Favorable SNPs of *HSP* 70 in Nigerian *B*. *indicus* can be used as a molecular biomarker for screening and selection of a large population of Nigerian native cattle for better thermo-tolerance and adaptability under thermal assault.

## Limitation of the Study

The Northern part of Nigeria, where the study was conducted, is frost with logistic constraints such as Boko Haram terrorism, Islamic jihad/insurgency, banditry, and kidnapping which limited sampling. The present study was conducted with only 90 animals; future study using larger sample size to validate current results is therefore recommended.

## Data Availability Statement

The raw data supporting the conclusions of this article will be made available by the authors, without undue reservation.

## Ethics Statement

The animal study was reviewed and approved by Federal University of Agriculture Research Ethics Committee.

## Author Contributions

All authors listed have made a substantial, direct and intellectual contribution to the work, and approved it for publication.

## Conflict of Interest

The authors declare that the research was conducted in the absence of any commercial or financial relationships that could be construed as a potential conflict of interest.
